# A universal polyphosphate kinase: PPK2c of *Ralstonia eutropha* accepts purine and pyrimidine nucleotides including uridine diphosphate

**DOI:** 10.1007/s00253-020-10706-9

**Published:** 2020-06-04

**Authors:** Jennie C. Hildenbrand, Attila Teleki, Dieter Jendrossek

**Affiliations:** 1 Institute of Microbiology, University of Stuttgart, Allmandring 31, 70569 Stuttgart, Germany; 2 Institute of Biochemical Engineering, University of Stuttgart, Stuttgart, Germany

**Keywords:** Polyphosphate, Polyphosphate kinase, ATP regeneration, Ralstonia eutropha

## Abstract

**Abstract:**

Polyphosphosphate kinases (PPKs) catalyse the reversible transfer of the γ-phosphate group of a nucleoside-triphosphate to a growing chain of polyphosphate. Most known PPKs are specific for ATP, but some can also use GTP as a phosphate donor. In this study, we describe the properties of a PPK2-type PPK of the β-proteobacterium *Ralstonia eutropha*. The purified enzyme (PPK2c) is highly unspecific and accepts purine nucleotides as well as the pyridine nucleotides including UTP as substrates. The presence of a polyP primer is not necessary for activity. The corresponding nucleoside diphosphates and microscopically detectable polyphosphate granules were identified as reaction products. PPK2c also catalyses the formation of ATP, GTP, CTP, dTTP and UTP from the corresponding nucleoside diphosphates, if polyP is present as a phosphate donor. Remarkably, the nucleoside-tetraphosphates AT(4)P, GT(4)P, CT(4)P, dTT(4)P and UT(4)P were also detected in substantial amounts. The low nucleotide specificity of PPK2c predestines this enzyme in combination with polyP to become a powerful tool for the regeneration of ATP and other nucleotides in biotechnological applications. As an example, PPK2c and polyP were used to replace ATP and to fuel the hexokinase-catalysed phosphorylation of glucose with only catalytic amounts of ADP.

**Key Points:**

• *PPK2c of R. eutropha can be used for regeneration of any NTP or dNTP.*

*• PPK2c is highly unspecific and accepts all purine and pyrimidine nucleotides.*

*• PPK2c forms polyphosphate granules* in vitro *from any NTP.*

**Electronic supplementary material:**

The online version of this article (10.1007/s00253-020-10706-9) contains supplementary material, which is available to authorized users.

## Introduction

Polyphosphate (polyP) is an inorganic linear polymer of phosphate residues linked by energy-rich phosphoanhydride bonds. PolyP can be formed either abiotically (e.g. by heating of phosphate-containing solutions in volcanic events) or biologically by the action of polyP kinases (PPKs). PolyP has been identified in all species that have been looked at and this is the basis for the assumption that polyP is a universal compound present in all living species on earth (Kornberg et al. 1999; Rao et al. 2009; Kulakovskaya and Kulaev 2013; Kulakovskaya et al. 2014). PolyP has a variety of functions in organisms: In prokaryotes, polyP is present in form of ≈ 100 to 200-nm-sized polyP granules (previously termed volutin granules) which are considered as reservoir for phosphorus and energy. Furthermore, polyP can be involved in various forms of stress resistance such as tolerance against heavy metals, elevated temperature, or reactive oxygen species. PolyP can be important for virulence, motility, biofilm formation, and cell cycle control as evident from analysis of polyP-deficient mutants (Rashid and Kornberg 2000; Rashid et al. 2000; Nikel et al. 2013; Chuang et al. 2013; Gray and Jakob 2015; Racki et al. 2017; Sultana et al. 2020). In humans, polyP plays a role not only in blood coagulation in platelets but is also involved in neurodegenerative diseases (Cremers et al. 2016; Lempart et al. 2019).

PolyP in prokaryotes is formed by polyP kinases (PPKs) (Rao et al. 2009; Kulakovskaya and Kulaev 2013). All PPKs catalyse the (reversible) formation of polyP from ATP by transferring the γ-phosphate group of ATP to a growing chain of polyP. Some PPKs can also use GTP for the formation of polyP or use GDP and polyP for the formation of GTP (see below). Two types of PPKs are known in bacteria: PPKs of the PPK1 type are ≈ 80 kDa proteins and consist of N, H, C1 and C2 domains (Zhu et al. 2005). PPKs of the PPK2 type are considerably smaller in molecular mass (MW around 40 kDa). Three subtypes of PPK2s are differentiated dependent on their substrate specificities for nucleoside monophosphates, nucleoside diphosphates or both (Motomura et al. 2014). Bacteria differ in whether they have only the PPK1 type of PPKs, only the PPK2 type of PPKs or both types of PPKs (PPK1 and PPK2) (Rao et al. 2009). The first characterized PPK was the one from *Escherichia coli* and belongs to the PPK1 family (Ahn and Kornberg 1990). The Kornberg laboratory also described the properties of the first PPK2 enzyme (from *Pseudomonas aeruginosa*) that revealed a high specificity for guanine nucleotides (Ishige et al. 1998, 2002; Zhang et al. 2002). Recently, it was shown that some PPK2 are able to form poly-phosphorylated nucleotides from polyP and ADP such as adenosine tetraphosphate [AT(4P)] or even adenosine-pentaphosphate [AT(5P)] in addition to ATP (Mordhorst et al. 2019).

*Ralstonia eutropha* (*Cupriavidus necator*) is well-known for its ability to accumulate poly(3-hydroxybutyrate) (PHB) in form of PHB granules during unbalanced growth conditions (Anderson and Dawes 1990; Madison and Huisman 1999; Pohlmann et al. 2006). The bacterium also forms polyP granules, and the genome sequence predicts the astonishing high number of seven PPK genes. Indeed, two PPK1s (PPK1a and PPK1b) as well as five PPK2s (PPK2a – PPK2d) have been identified in *R. eutropha* (Tumlirsch et al. 2015). One of the PPK2s, PPK2c, turned out to be highly active and catalysed the formation of microscopically detectable polyP granules in vitro, if ATP or GTP was present as a substrate (Hildenbrand et al. 2019). In this study, we determined the biochemical properties of PPK2c and investigated the substrate specificity for different nucleoside-phosphates.

## Material and methods

### Bacterial strains, plasmids and culture conditions

In this study, *Escherichia coli* JM109 and *E. coli* BL21(DE3)/pLysS (Novagen) were used for cloning procedures and gene expression, respectively. Cloning of the *ppk2c* gene into the pET28a expression vector was done with *Nde*I and *Eco*RI as restriction sites. The pET28a-*ppk2c* construct was transformed into *E. coli* JM109, verified by PCR amplification and sequencing, before finally transformed into the expression strain *E. coli* BL21(DE3)/pLysS.

### Purification of PPK2c

The overexpression of *ppk2c* and purification of PPK2c with an N-terminal hexa-histidine tag was done as previously described (Hildenbrand et al. 2019). Purified PPK2c (1.5 mg/ml) was shock-frozen in 0.2-ml aliquots in liquid nitrogen and stored at − 70 °C.

### Fluorescence microscopic detection of PPK2c-formed polyP

Formation of polyP granules was followed by fluorescence microscopy after staining polyP with DAPI solution (0.1 mg/mL in distilled H_2_O) for at least 10 min and was performed as it has been described in our previous publication (Hildenbrand et al. 2019) with the exception that samples had been taken after different time points.

### PPK activity assay in the direction of polyP formation

Assays were performed in 15-μl assay buffer consisting of 0.1-M Tris-HCl (pH 8.0), 2- or 5-mM MnCl_2_, 1-mM NTP (ATP, GTP, CTP, dTTP or UTP, for HPLC assay or for fluorescence microscopy; 15-mM NTPs for polyP detection by gel electrophoresis) and 15–μM of purified PPK2c enzyme. Since TTP was not commercially available, desoxyTTP (dTTP) was used instead. In some experiments, no MnCl_2_ was added or MnCl_2_ was replaced by 2-mM MgCl_2,_ CaCl_2_ or ZnCl_2_. Samples were incubated at 30 °C with constant shaking at 450 rpm for 30 min. The reaction was stopped by heating the solution at 95 °C for 2 min in an Eppendorf incubator, followed by a centrifugation step at 12,000 rpm for 3 min at room temperature (RT) to remove precipitated protein. Ten μl of the supernatant was either transferred to a HPLC reaction vial or stained with DAPI and subsequently spotted on an agarose gel pad (1%, in distilled water) for fluorescence microscopy.

### PPK activity assay in the direction of polyP consumption

Assays were performed in 15-μl assay buffer consisting of 0.1-M Tris-HCl (pH 8.0), 2- or 5-mM MnCl_2_, 0.5–2-mM NDP (ADP, GDP, CDP, dTDP or UDP), 9-mM polyP (referred to phosphate content) with an average length of ≈ 100-P_i_ residues and 1 μM of purified PPK2c enzyme. Samples were incubated at 30 °C with constant shaking at 450 rpm for 30 min. The reaction was stopped by heating the solution at 95 °C for 2 min in an Eppendorf incubator, followed by a centrifugation step at 12,000 rpm for 3 min at room temperature (RT) to remove precipitated protein. Ten μl of the supernatant was transferred to an HPLC vial for detection of nucleoside phosphates.

### Hexokinase reaction in the absence of added ATP

The assay was conducted at 25 °C in 1-ml quartz cuvettes containing 877-μl 0.1-M Tris-HCl, pH 7.7, 20–μl of 100-mM MgCl_2_, 5 μl of 100-mM ADP, 10 μl of 100-mM NADP, 10 μl of 100-mM glucose, 2 μl of purified PPK2c (35 μM) and 10 μl of a hexokinase/glucose-6-phosphate dehydrogenase mixture (3 mg/ml). The reaction was started by the addition of 50-μl polyP (100 mM, referred to monomeric phosphate) and followed at 340 nm using a Cary 100 UV-Vis spectrophotometer (Agilent).

### Detection of nucleoside phosphates via high performance liquid chromatography (HPLC)

Samples were analysed by using a reverse phased HPLC column (ISAspher 100–5 C18 BDS 250 × 4.0-mm column) on a HPLC instrument (Infinity, Agilent) with a detection wavelength for product peaks of 254 nm. The buffers used as the mobile phase were buffer A with 0.1-M potassium phosphate, 4-mM tetrabutylammonium hydrogensulphate (Sigma-Aldrich) (pH 6.0) and buffer B consisting of 40% methanol and 60% buffer A (pH 7.2). Flow rate and column temperature were set to 0.9 ml min^−1^ and 20 °C, respectively. The sample injection volume was 3 μl. Starting with 70% buffer A and 30% buffer B, gradient elution was carried out by applying 30–100% B for 10 min, followed by 100–30% B for 7 min. Using these conditions, the various nucleoside-phosphates eluted at times as summarized in Table 1. Online Resource 1 shows HPLC chromatograms for pure nucleoside di- and triphosphates.

**Table 1:** Nucleotides investigated in this study

Nucleotide	Retention time [min]
AMP	4.7
ADP	6.4
ATP	7.8
AT(4)P*)	8.2
GMP	3.1
GDP	3.7
GTP	4.8
GT(4)P*)	5.9
CMP	2.6
CDP	3.0
CTP	3.7
CT(4)P*)	4.7
dTMP	4.6
dTDP	5.5
dTTP	7.1
dTT(4)P*)	7.7
UMP	2.9
UDP	3.5
UTP	4.8
UT(4)P*)	6.1

The retention times of the nucleotides in the HPLC-assay are provided. Depending on the batch and the age of the HPLC column, the retention times varied up to 0.3 min. However, within one batch of HPLC runs, the deviation in retention times was always ≤ 0.1 min

*) validated by HPLC-MSi/MS

### Detection of nucleoside phosphates via HPLC-tandem mass spectrometry (HPLC-MS/MS)

For identification of the PPK2c reaction products by HPLC/MS, the following assay was performed as follows: 1-μM PPK2c enzyme was incubated in 0.1-M Tris-HCl buffer (pH 8.0) containing 0.5-mM NDP’s, 3-mM MnCl_2_ and 9-mM polyP at 30 °C. After 30 min, the sample was shock-frozen with liquid nitrogen and stored at − 70 °C until measurement. Samples were thawed on ice and ultra-filtrated in Roti-Spin MINI-3 columns (3 kDa cut-off) by centrifugation (15 min) at 20,000 g and 4 °C. LC-MS/MS measurements were performed on an Agilent 1200 HPLC system coupled with an Agilent 6410B triple quadrupole mass spectrometer (MS-QQQ) with an electrospray ionization (ESI) ion source. The chromatographic separation of nucleoside phosphates was performed by bicratic polymer-based zwitterion hydrophilic interaction chromatography (ZIC-pHILIC) under alkaline mobile phase conditions (Feith et al. 2019). Mixtures of nucleoside phosphate standards or filtered assay samples each containing 25-μM 2-amino-N-isopropyl benzamide (AIBA) as internal standard or filtered assay samples (see Table 2) were injected (5 μl) onto a Sequant ZIC-pHILIC column (150 × 2.1 mm, 5 μm, Merck Millipore) equipped with a guard column (20 × 2.1 mm, 5 μm, Merck Millipore) maintained at 40 °C. Mobile phases (constant flow rate of 0.2 ml min^−1^) were composed of aqueous buffer solutions (10-mM ammonium acetate, pH 9.2) with 90% (v/v) acetonitrile for eluent A and 10% (v/v) acetonitrile for eluent B, using the following program for gradient elution: isocratic hold 0% B for 1 min, linear gradient from 0% B to 75% B for 30 min, linear gradient from 75% B to 100% B for 4 min, isocratic hold 100% B for 5 min, linear gradient from 100% B to 0% B for 10 min and equilibration to starting conditions by an isocratic hold 0% B for 15 min. ESI source parameters were set as follows: nitrogen gas flow rate of 10 L/min at 350 °C, capillary voltages of ± 4.0 kV and a nebuliser pressure of 30 psi. Nucleoside mono-, di- and triphosphates were detected in negative ionization mode (ESI-) with high selectivity in the multiple reaction monitoring (MRM) mode based on pre-optimized precursor-to-product ion transitions with a mass resolution of 0.1 u and associated MS/MS parameters (Teleki et al. 2015). Corresponding nucleoside-tetraphosphates were detected by calculated precursor-to-product ion transitions and transferred MS/MS parameters (Online Resource 2). System control, acquisition and data analysis were obtained by using commercial MassHunter B.07.00 software.

**Table 2:** Sample preparation for HPLC-MS/MS

Component	Volume [μl]	Dilution factor
Acetonitrile (ACN)	60	1.7
1-M NH_4_-acetate/pH 9.2	1	100
sample	30	3.4
2.5-mM AIBA	1	100
Water	8	12.5
Total	100	

The detection and identification of nucleotides by HPLC-MS/MS was performed by applying 5-μl sample injections of the respective assay mixture

### Toluidine-staining of polyP after polyacrylamide gel electrophoresis

PolyP formation assays were performed in 100-μl assay buffer consisting of 0.1-M Tris-HCl (pH 8.0), 2-mM MnCl_2_, 15-mM NTP (ATP, GTP, CTP, dTTP or UTP) and 5-μM PPK2c enzyme. Samples were incubated at 30 °C with constant shaking at 450 rpm for 150 min. The reaction was stopped by heating the solution at 95 °C for 2 min in an Eppendorf incubator, followed by a centrifugation step at 12,000 rpm for 3 min at room temperature (RT) to remove precipitated protein. Enzymatically produced polyP was then detected by toluidine blue staining after electrophoretic separation in a polyacrylamide gel (15%) according to (Losito et al. 2009).

## Results

### PPK2c is highly active in vitro

**Fig. 1:**
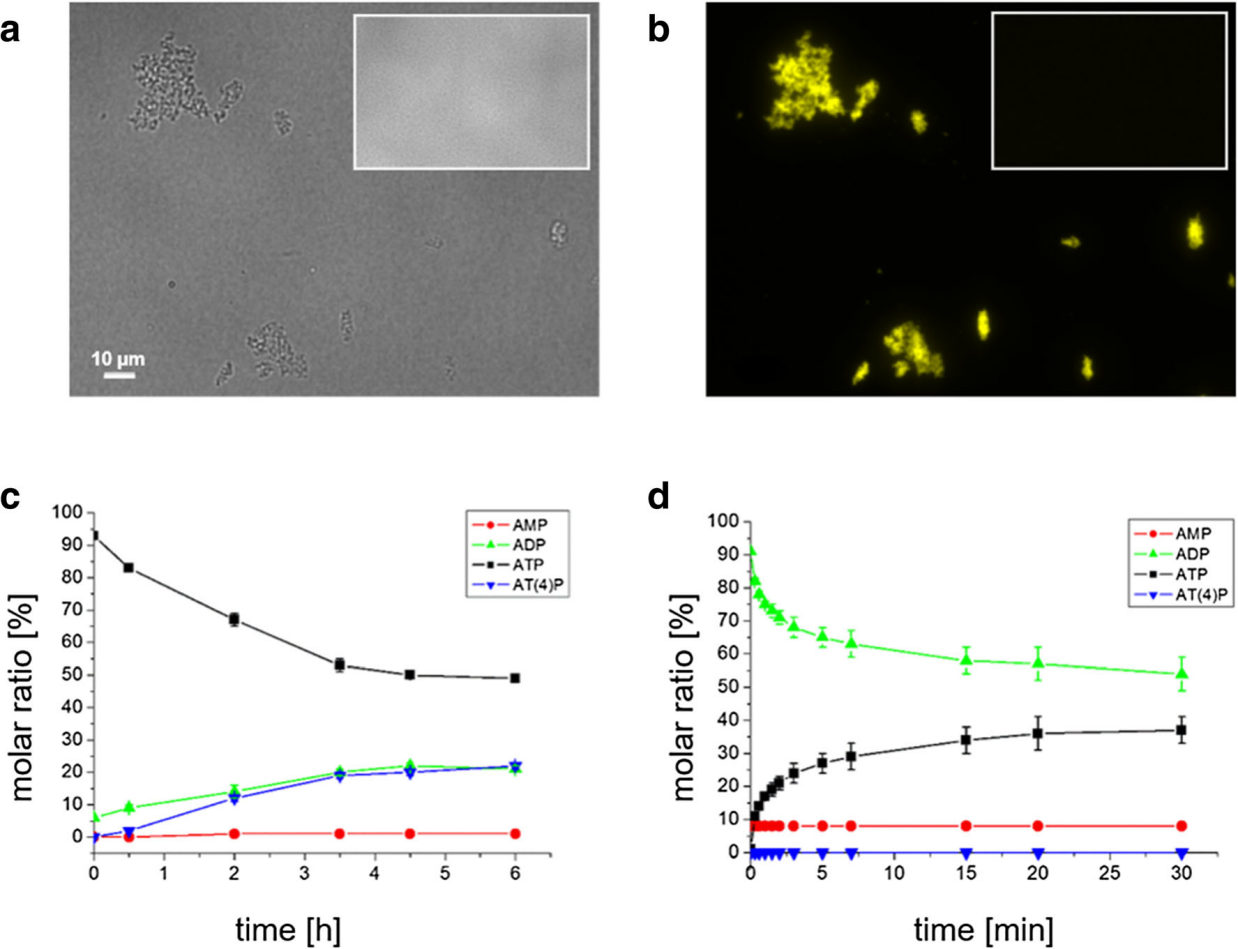
In vitro formation of polyP by PPK2c. Purified PPK2c was incubated with 15-mM ATP at 30 °C and subsequently stained with DAPI. Globule-like aggregates were detected in bright field (**a**) that showed DAPI-polyP specific fluorescence (**b**). The inlay images show controls in which no PPK2c was present at otherwise same conditions. HPLC-activity assay of purified PPK2c in the direction of polyP synthesis from ATP (**c**) and in the direction of ATP synthesis from polyP (**d**). Assays were performed in triplicate; error bars indicate standard deviations

PPK2c with an N-terminal hexa-histidine tag was purified and stored frozen in 50-mM Tris-HCl (pH 8.0), 500-mM NaCl, 100-mM imidazole and 20% glycerol, as described in the method section. The presence of NaCl was necessary to prevent precipitation of PPK2c. The enzyme was highly active as revealed by the formation for microscopically detectable polyP granules that could be stained with DAPI and imaged at a DAPI-polyP specific emission wavelength (Fig. 1a, b). Control experiments without enzyme, with heat-inactivated PPK2c, or without ATP resulted in no formation of detectable polyP (inlays in Fig. 1a, b and Online Resource 4). The reaction was rather rapid as polyP granules could be detected already after only 1 min reaction time (data not shown). For quantitative analysis, we determined the activity of PPK2c by HPLC: When PPK2c was assayed in the direction of polyP formation from ATP (Fig. 1c, an almost linear decrease of the ATP concentration was measured for up to 3 h of the assay, and a value of 42-nmol consumed ATP min^−1^ x mg^−1^ protein was determined. The reverse reaction of PPK2c, i.e. in the formation of ATP from polyP and ADP, was much more rapid. As shown in Fig. 1d, a rapid decrease of the ADP concentration was accompanied by an equivalent rapid increase of the ATP concentration. The reaction slowed down within minutes and the concentrations of ADP and ATP approximated to each other after ≈ 30 min reaction time. A value of 3.6-μmol consumed ADP min^−1^ × mg^−1^ protein was calculated from the initial phase of the reaction (0–20 s). The same value was calculated from the increase of the ATP concentration.

### Optimal assay conditions of PPK2c

**Fig. 2:**
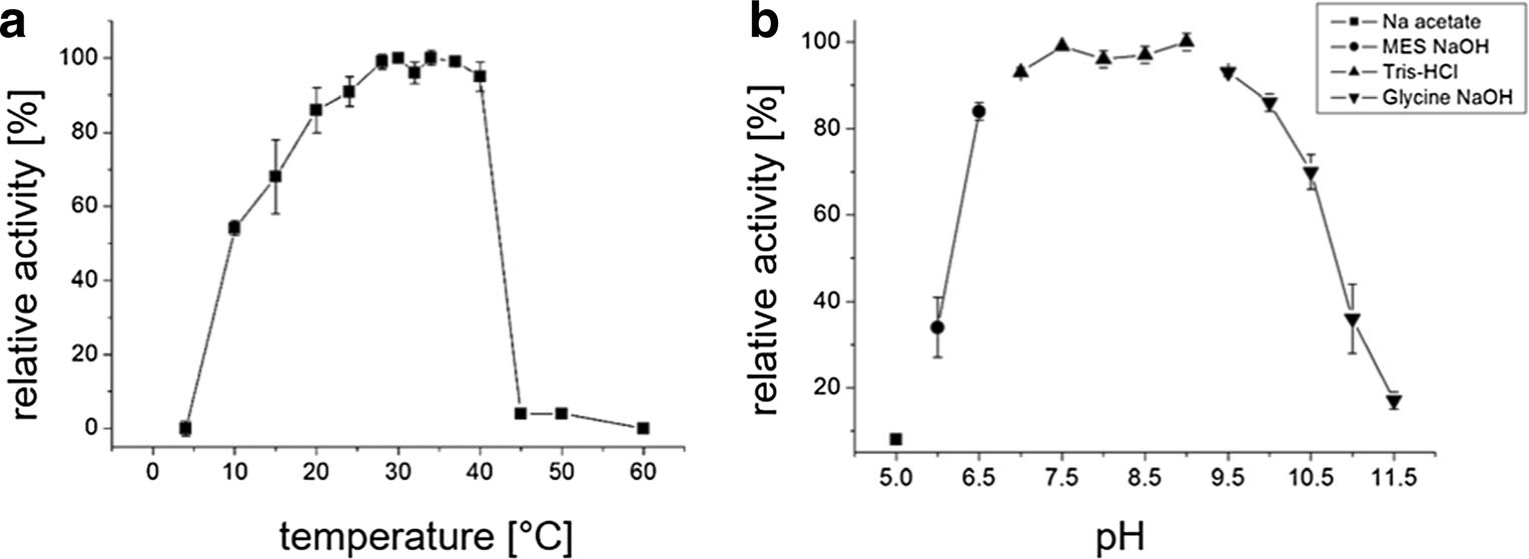
Temperature and pH optimum of PPK2c. The dependence of PPK2c activity (direction of ATP formation from polyP) on temperature (**a**) and pH (**b**) was determined using the HPLC-based assay. The 100% values in (**a**) and (**b**) correspond to 1.2 μmol/min/mg, respectively (ADP consumption). Assays were performed in triplicate; error bars indicate standard deviations

PPK2c activity was determined at different temperatures and different pH values using the HPLC assay (Fig. 2a, b). It turned out that PPK2c showed highest activity between ≈ 25 and 40 °C and between pH 6.5 and 10. At temperatures above 40 °C, PPK2c was rapidly inactivated.

### PPK2c needs magnesium or manganese ions for activity

**Fig. 3:**
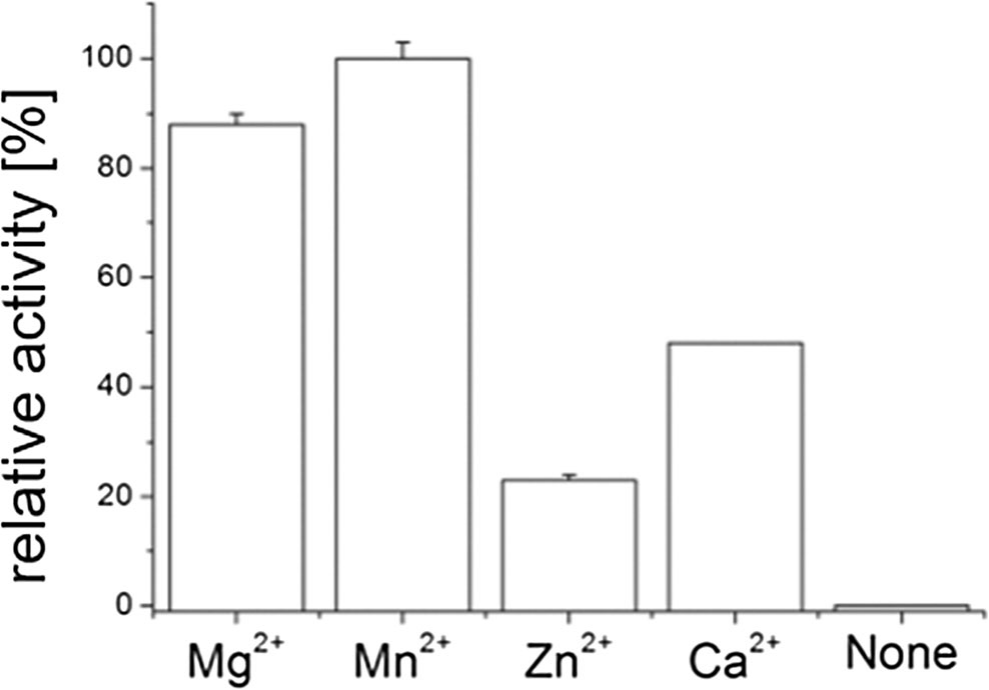
Dependence of PPK2c activity from divalent cations. PPK2c activity assays (direction of ATP formation from polyP and ADP) were performed in the absence or presence of 5 mM of cations as indicated. The highest activity (1.1 μmol/min/mg ADP consumption, expressed as average of the 30 min assay period) was determined when Mn^2+^ was used as a cofactor and was taken as 100%. Assays were performed in triplicate; error bars indicate standard deviations

Next, we tested the dependence of PPK2c activity from divalent cations (Fig. 3). Hardly any activity was determined in the absence of divalent cations. Highest activity was determined when 5-mM MnCl_2_ was present; MgCl_2_ was almost as good as manganese salts (84% activity). The activities were substantially lower with calcium or zinc salts (48 or 21%, respectively).

### PPK2c utilizes purine and pyrimidine nucleoside triphosphates including uridine-triphosphate for polyP formation

**Fig. 4:**
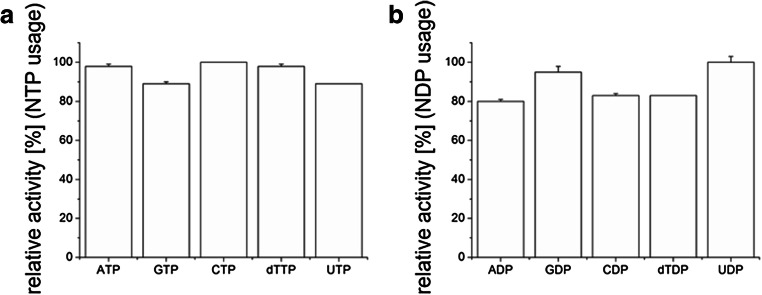
Substrate specificity of PPK2c. PPK2c was incubated with either nucleoside triphosphates (**a**) or with nucleoside diphosphates and polyP (**b**) and the consumption of the respective substrates was determined. All assays were performed with 2-mM nucleoside phosphates and 1-μM PPK2c and were run for 3.5 h (**a**) or 30 min (**b**) at 30 °C. The highest activity in (**a**) (0.1 μmol/min/mg expressed as average of the 3.5 h assay period) was determined with CTP and was taken as 100%. For (**b**), the highest activity was measured with UDP (1.3 μmol/min/mg) and was taken as 100%. Assays were performed in triplicate; error bars indicate standard deviations

**Fig. 5:**
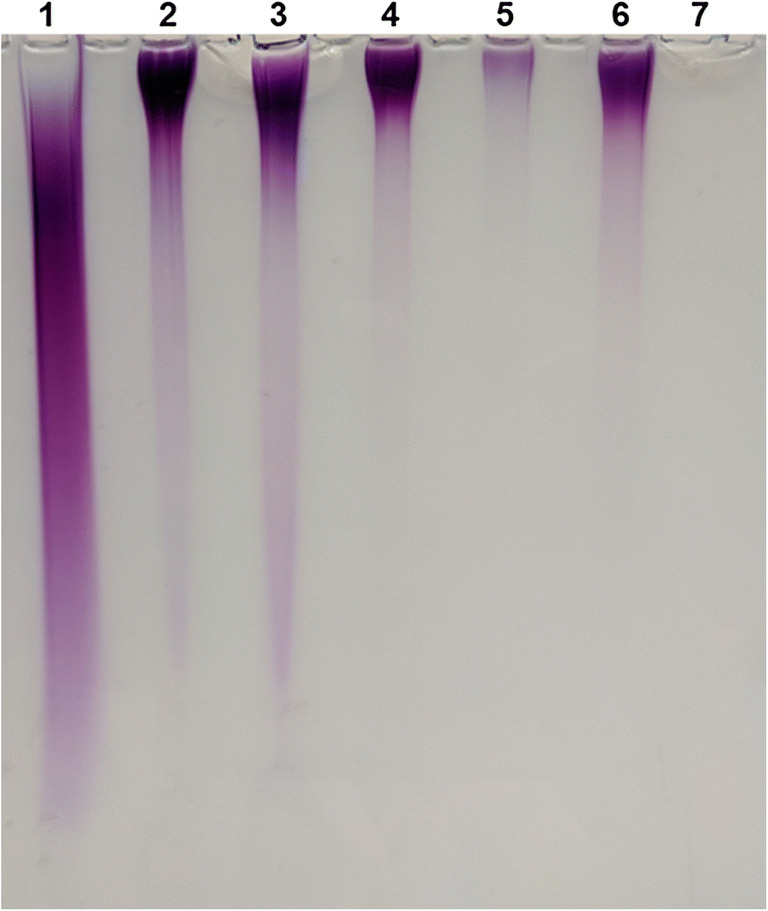
Separation of PPK2c-produced polyP by PAGE and subsequent toluidine staining. Standard polyP (average chain length ≈ 100 Pi residues corresponding to 10-mM P_i_) (lane 1), 15 mM of ATP, GTP, CTP, dTTP or UTP (lanes 2–6), control with 15-mM ATP but without PPK2c (lane 7)

All currently known PPKs are able to utilize ATP for polyP synthesis (Ishige et al. 2002; Motomura et al. 2014). However, for most PPKs it is not known whether and to which extent they can utilize other substrates. We therefore tested adenine, guanine, cytosine, thymidine and uridine nucleoside di- and triphosphates as potential substrates for PPK2c. As shown in Fig. 4a, b and Online Resource 3, PPK2c is very unspecific and converted all tested purine and pyrimidine nucleoside phosphates at comparable rates. A nucleotide-related compound, thiamine pyrophosphate was, however, not used as a substrate by PPK2c (data not shown). When nucleoside triphosphates were used as substrates for the formation of polyP (at 1 mM), the addition of an oligophosphate primer was not necessary in any case. The formation of polyP was verified for both purine nucleoside triphosphates and all three pyrimidine nucleoside triphosphates (and by all dNTPs, data not shown for dATP, dGTP, dCTP) by fluorescence microscopic detection of DAPI-stainable polyP granules (Online Resource 4). Furthermore, the chain length of the formed polyP molecules from the different nucleoside triphosphates was evaluated by PAGE and subsequent staining with toluidine blue (Fig. 5). Regardless of the type of nucleoside triphosphates, high molecular weight polyP was formed. Most of the PPK2c-formed polyP molecules migrated much less into the polyacrylamide gel compared with the polyP standard. Since the average number of phosphate residues of the polyP standard is roughly around 100, it is evident that most polyP molecules made by PPK2c have a considerable higher chain length. However, in case of ATP and GTP as substrate, low chain length polyP molecules were also detected.

### PPK2c catalyses the formation of nucleoside tetraphosphates

**Fig. 6:**
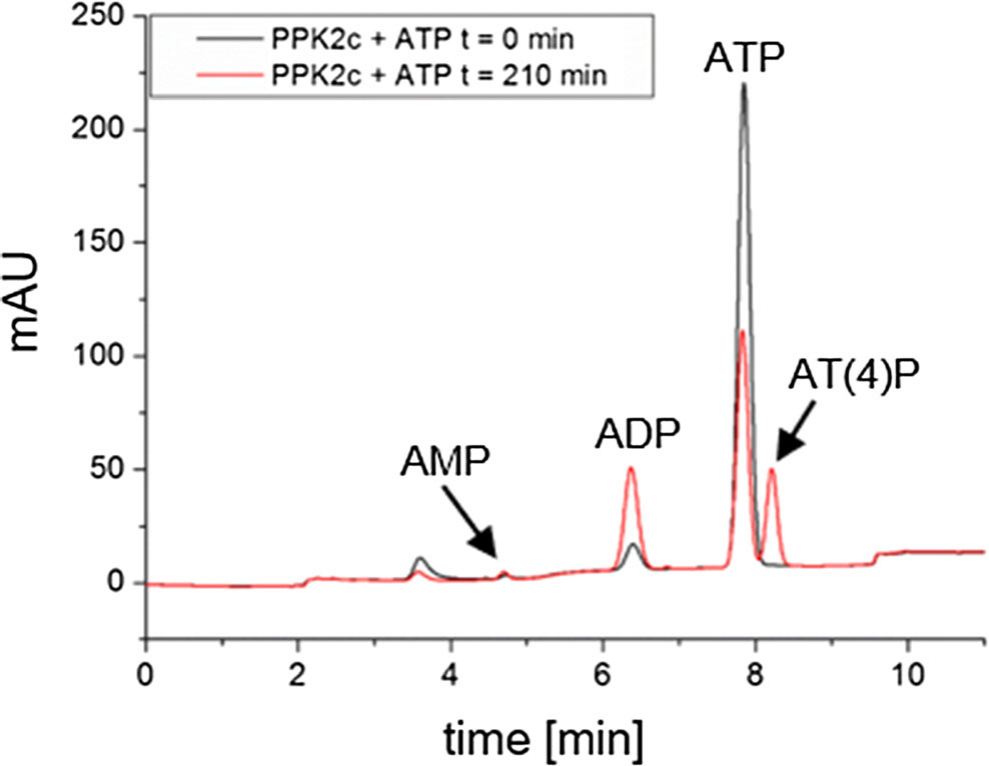
In vitro activity of PPK2c with ATP as substrate. The concentrations of adenosine nucleotides were determined before (0 min, black) and after the addition of PPK2c (210 min, red) by HPLC. (mAU, milli-absorption units at 254 nm)

Interestingly, an additional peak was observed at higher retention times than ATP in chromatograms of reactions when PPK2c was assayed (Fig. 6). Similar additional peaks at higher retention times than the respective nucleoside triphosphates were detected when the assay was performed with GTP, CTP, dTTP or UTP. These additional peaks were also detected at the same retention times (although in lower intensity) when the reaction was performed in the direction of nucleoside triphosphate synthesis from polyP (see Online Resource 3 for overview of all reaction products). To determine the masses of the compounds behind these unexpected peaks, HPLC-ESI/MS of the reaction products of PPK2c was performed. Preliminary direct injection analyses (column bypassing with *m/z* full scan) of PPK2c assays enable the detection of additional [M-H]^−^ precursor masses in negative ionization mode with *m/z* values of 586, 602, 562, 561 and 563 if ADP, GDP, CDP, dTDP or UDP were used as substrate, respectively. Determined values each differed by 80 Da from the corresponding nucleoside triphosphate masses. Targeted QQQ-MS/MS measurements with corresponding MRM mass transitions using phosphate ester residues as [M-2H]^−^ fragment ions (*m/z* 79) confirmed the additional formation of nucleoside tetraphosphates, namely, adenosine-tetraphosphate (AT(4)P), guanosine-tetraphosphate (GT(4)P), cytidine-tetraphosphate (CT(4)P), desoxy-thymidine-tetraphosphate (dTT(4)P) and uridine-tetraphosphate in all five samples (Online Resources 2 and 5). These findings correspond to the previous identification of AT(4)P as an additional reaction product of the PPK of *E. coli* (Ahn and Kornberg 1990), recently also found for PPK2s of *Meiothermus ruber* (Mordhorst et al. 2019) and of *Delftia tsuruhatensis* (Ogawa et al. 2019). It should be noted that the nucleoside tetraphosphates were only formed when manganese was present in the assay buffer and were not detected in assays with magnesium salts.

### PPK2c belongs to subgroup I of type 2 PPKs

PPKs are divided into three subgroups depending on whether they phosphorylate nucleoside diphosphates (subgroup I), nucleoside monophosphates (subgroup II) or both (subgroup III) (Motomura et al. 2014). To identify the subgroup type of PPK2c, we tested the ability of PPK2c to phosphorylate nucleoside monophosphates in the presence of polyP. However, nucleoside diphosphates were never formed by PPK2c regardless which type of purine or pyrimidine nucleoside monophosphate was used suggesting that PPK2c is a member of subgroup I. We also tested the ability of PPK2c to form polyP from ADP (in the absence of ATP). However, no reaction products (AMP or polyP) could be detected.

### Utility of PPK2c to replenish ATP in ATP-consuming biochemical reactions

**Fig. 7:**
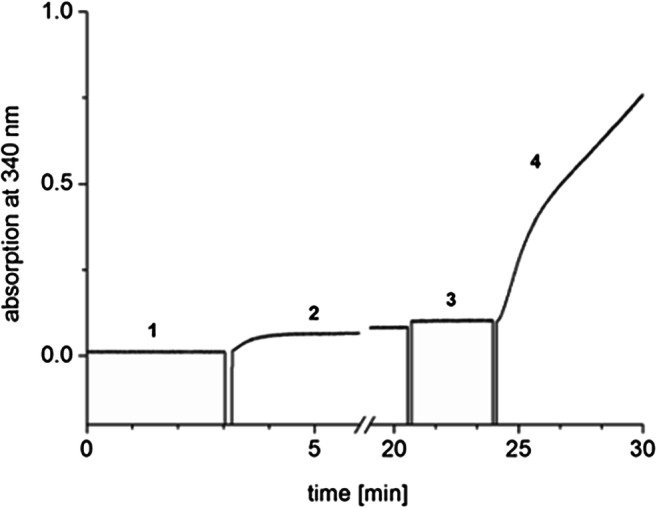
PolyP-dependent generation of ATP for glucose phosphorylation in a coupled hexokinase/glucose-6-phosphate dehydrogenase (G6P-DH) assay. The reaction was performed in a 1-ml quartz-cuvette at 25 °C. Measurements started with preincubation of hexokinase/G6P-DH mix in Tris-HCl buffer, pH 7.7, containing 2-mM MgCl_2_, together with 1-mM NADP and 1-mM glucose (phase 1). After incubation for 3 min, 0.5-mM ADP was added which resulted in a slight increase of absorbance at 340 nm due to a contamination of ADP with ATP (phase 2) (see also Online Resource 1). At minute ≈ 21, PPK2c was added to the mixture (phase 3). The reaction was started by the addition of 5-mM polyP (at ≈ 24 min) and led to a rapid increase of the absorption signal (NADPH production, phase 4) indicating that glucose-6-phosphate had been formed and was used for the G6P-DH reaction. Controls without ADP resulted in no reaction

To investigate the utility of PPK2c as an auxiliary enzyme, we exemplarily tested PPK2c for its ability to replenish ATP in a hexokinase reaction. To this end glucose was incubated with ADP (instead of ATP), polyP, PPK2c and hexokinase. For monitoring the reaction, the formed glucose-6-phosphate was oxidized in the presence of NADP by the helper enzyme, NADP-dependent glucose-6-phosphate dehydrogenase, to 6-phosphogluconate and NADPH as shown in Fig. 7. The strong reaction (NADPH generation) showed that despite the absence of added ATP (only ADP was present) glucose was phosphorylated by hexokinase emphasizing the utility of PPK2c as an auxiliary enzyme to refill ATP pools on the expense of polyP in biochemical reactions. Since PPK2c is very unspecific and accepts all purine and pyrimidine nucleoside phosphates, it will be the enzyme of choice even if nucleoside triphosphates other than ATP are used or if the nucleoside phosphate specificity of an enzymatic reaction in a non-defined system (crude extracts) is not known.

## Discussion

Despite an increasing number of reports on the properties of PPKs and on the utility of PPKs for the regeneration of ATP in ATP-requiring reactions (for recent examples see (Kamatani et al. 2018; Ogawa et al. 2019; Zhang et al. 2020; Wang et al. 2020)), only few PPKs have been tested for their specificity towards pyrimidine nucleotides. The first one is the PPK of *E. coli* (a PPK1 type enzyme that prefers ADP over GDP and GDP over UDP and CDP (Kuroda and Kornberg 1997)) and the other is PPK2 of *Meiothermus ruber*. The PPK of this species is able to utilize the pyrimidine nucleotides CTP and UDP in addition to ATP and GTP (Motomura et al. 2014). A nucleoside diphosphate kinase activity of a PPK for thymidine phosphates to our knowledge has not yet been described. Our study showed that PPK2c of *R. eutropha* utilizes all natural nucleotides as substrates at comparable activities and in vitro works in both directions (Fig. 4). Therefore, PPK2c is the most unspecific (and thus universal) PPK that is presently known. Due to its easy production in recombinant *E. coli* in large quantities, its high activity and stability during storage as well as its broad pH range for activity, we propose PPK2c of *R. eutropha* to become the enzyme of choice in biotechnological applications when the regeneration of nucleoside triphosphates is necessary.

The presence of seven *ppk* genes in the *R. eutropha* genome is surprising. Many other bacteria have only one or two *ppk* genes. This raises the question for the benefit of having seven *ppk* genes. Since mutants deficient in *ppk2c* still are able to form polyP granules, PPK2c is not essential for polyP formation (Tumlirsch et al. 2015). It is known that PPK2s can have a broad substrate specificity and can utilize guanine nucleotides in addition to adenine nucleotides (Zhang et al. 2002; Ishige et al. 2002) in comparison with PPK1s. This suggests that PPK2s could have a function in the regeneration of GTP. In our study, we showed that PPK2c is able to accept any natural occurring nucleotide as a substrate including pyridine nucleotides. Based on the high activity of PPK2c with the desoxynucleotides and with nucleotides, PPK2c is able to utilize both DNA and RNA nucleotides. Therefore, we think that PPK2c may have the physiological function to replenish any nucleoside- and desoxynucleoside-triphosphate pools in times of enhanced demand on the expense of previously accumulated polyP. In particular, the replication of the genome and high transcriptional activities require the availability of desoxynucleotides and nucleotides, respectively. The substrate specificities, biochemical properties and putative physiological functions of the six other PPKs in *R. eutropha* (PPK1a, PPK1b, PPK2a, PPK2b, PPK2d and PPK2e, (Tumlirsch et al. 2015)) remain to be determined.

## Electronic supplementary material

ESM 1 (PDF 3389 kb).
