# Supplementary material for: A universal polyphosphate kinase: PPK2c of Ralstonia eutropha accepts purine and pyrimidine nucleotides including uridine diphosphate
Source: Appl Microbiol Biotechnol. 2020 Jun 4;104(15):6659–67. doi: 10.1007/s00253-020-10706-9 (PMC7347700; doi:10.1007/s00253-020-10706-9)
Supplement: Supplementary file 1 — (PDF 3389 kb). [file 253_2020_10706_MOESM1_ESM.pdf]

Applied Microbiology and Biotechnology

Online Resources

A universal polyphosphate kinase: PPK2c of *Ralstonia eutropha* accepts purine and pyrimidine nucleotides including uridine diphosphate

Jennie C. Hildenbrand[1], Attila Teleki[2], and Dieter Jendrossek[1*]

[1]*Institute of Microbiology, University of Stuttgart, Germany*

[2]*Institute of Biochemical Engineering, University of Stuttgart, Germany*

*Correspondent footnote: Dieter Jendrossek
Institut für Mikrobiologie
Universität Stuttgart
Allmandring 31
70569 Stuttgart
Germany
Tel.: +49-711-685-65483
Fax: +49-711-685-65725
Email: dieter.jendrossek@imb.uni-stuttgart.de

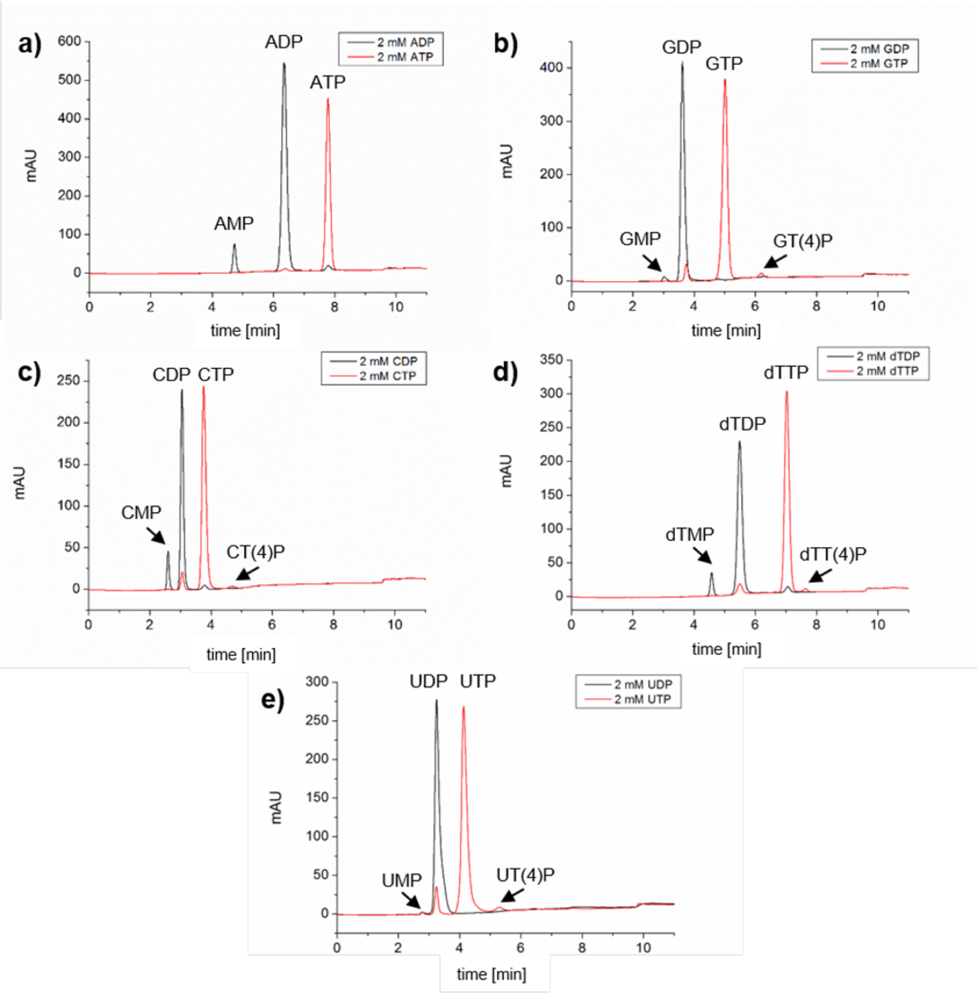

Online Resource 1: HPLC-separation of nucleoside di- and triphosphates (a) ADP and ATP, b) GDP and GTP, c) CDP and CTP, d) dTDP and dTTP, e) UDP and UTP) used as substrates for PPK2c. Traces of the corresponding nucleoside mono-phosphates were detected for the nucleoside diphosphate standards and traces of nucleoside tetraphosphates were detected in preparations of nucleoside triphosphate standards (except for ATP). (mAU, milli-absorption units at 254 nm).

Online Resource 2: HILIC-QQQ-MS/MS parameters for targeted measurements of nucleoside phosphate species

Compound	Abbr.	Sum Formula	ESI	Precursor		Fragment		QQQ-MS/MS (MRM)		HILIC
			Polarity	Species	(m/z)	Species	(m/z)	CE [V]	F [V]	tR [min]
**Adenosine-5'-monophosphate**	AMP	$C_{10}H_{14}N_5O_7P$	**[-]**	[M-H]-	**346**	[M-2H]-	**79**	60	135	**18.84**
**Adenosine-5'-diphosphate**	ADP	$C_{10}H_{15}N_5O_{10}P_2$	**[-]**	[M-H]-	**426**	[M-2H]-	**79**	98	135	**21.28**
**Adenosine-5'-triphosphate**	AT(3)P	$C_{10}H_{16}N_5O_{13}P_3$	**[-]**	[M-H]-	**506**	[M-2H]-	**79**	108	135	**23.26**
**Adenosine-5'-tetraphosphate**	AT(4)P	$C_{10}H_{17}N_5O_{16}P_4$	**[-]**	[M-H]-	**586**	[M-2H]-	**79**	108	135	**25.36**
**Guanosine-5'-monophosphate**	GMP	$C_{10}H_{14}N_5O_8P$	**[-]**	[M-H]-	**362**	[M-2H]-	**79**	60	135	**22.22**
**Guanosine-5'-diphosphate**	GDP	$C_{10}H_{15}N_5O_{11}P_2$	**[-]**	[M-H]-	**442**	[M-2H]-	**79**	98	135	**24.48**
**Guanosine-5'-triphosphate**	GT(3)P	$C_{10}H_{16}N_5O_{14}P_3$	**[-]**	[M-H]-	**522**	[M-2H]-	**79**	108	135	**26.31**
**Guanosine-5'-tetraphosphate**	GT(4)P	$C_{10}H_{17}N_5O_{17}P_4$	**[-]**	[M-H]-	**602**	[M-2H]-	**79**	108	135	**28.58**
**Cytidine-5'-monophosphate**	CMP	$C_9H_{14}N_3O_8P$	**[-]**	[M-H]-	**322**	[M-2H]-	**79**	60	135	**21.42**
**Cytidine-5'-diphosphate**	CDP	$C_9H_{15}N_3O_{11}P_2$	**[-]**	[M-H]-	**402**	[M-2H]-	**79**	98	135	**23.64**
**Cytidine-5'-triphosphate**	CT(3)P	$C_9H_{16}N_3O_{14}P_3$	**[-]**	[M-H]-	**482**	[M-2H]-	**79**	108	135	**25.51**
**Cytidine-5'-tetraphosphate**	CT(4)P	$C_9H_{17}N_3O_{17}P_4$	**[-]**	[M-H]-	**562**	[M-2H]-	**79**	108	135	**27.81**
**Thymidine-5'-monophosphate**	dTMP	$C_{10}H_{15}N_2O_8P$	**[-]**	[M-H]-	**321**	[M-2H]-	**79**	60	135	**17.63**
**Thymidine-5'-diphosphate**	dTDP	$C_{10}H_{16}N_2O_{11}P_2$	**[-]**	[M-H]-	**401**	[M-2H]-	**79**	98	135	**20.67**
**Thymidine-5'-triphosphate**	dTT(3)P	$C_{10}H_{17}N_2O_{14}P_3$	**[-]**	[M-H]-	**481**	[M-2H]-	**79**	108	135	**22.69**
**Thymidine-5'-tetraphosphate**	dTT(4)P	$C_{10}H_{18}N_2O_{17}P_4$	**[-]**	[M-H]-	**561**	[M-2H]-	**79**	108	135	**24.88**
**Uridine-5'-monophosphate**	UMP	$C_9H_{13}N_2O_9P$	**[-]**	[M-H]-	**323**	[M-2H]-	**79**	60	135	**20.41**
**Uridine-5'-diphosphate**	UDP	$C_9H_{14}N_2O_{12}P_2$	**[-]**	[M-H]-	**403**	[M-2H]-	**79**	98	135	**22.91**
**Uridine-5'-triphosphate**	UT(3)P	$C_9H_{15}N_2O_{15}P_3$	**[-]**	[M-H]-	**483**	[M-2H]-	**79**	108	135	**24.82**
**Uridine-5'-tetraphosphate**	UT(4)P	$C_9H_{16}N_2O_{18}P_4$	**[-]**	[M-H]-	**563**	[M-2H]-	**79**	108	135	**27.13**

tR, retention time of compound on HILIC column

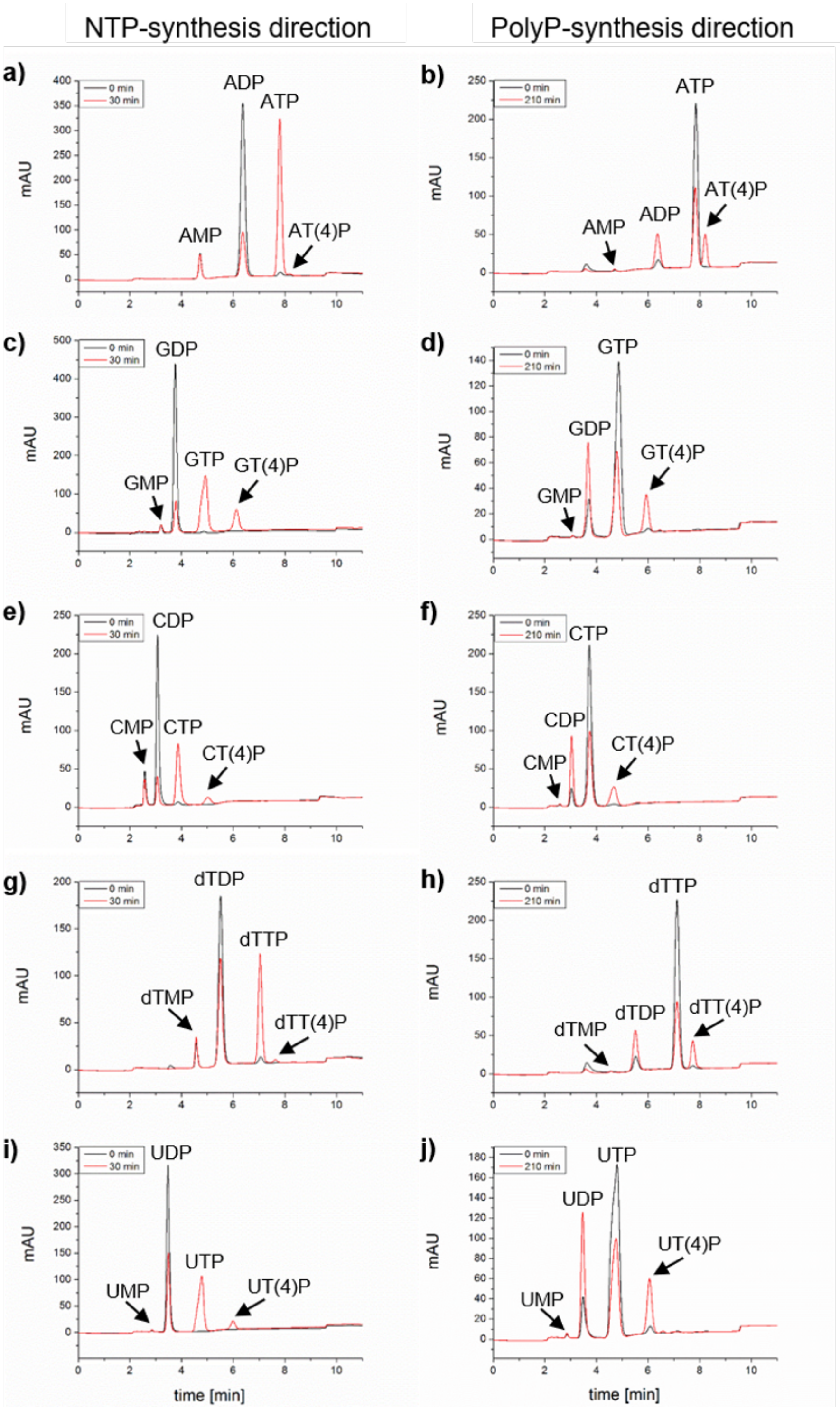

Online Resource 3: Educts and products of PPK2c catalysed reactions.
The concentrations of the different nucleoside phosphates were determined by HPLC before the addition of enzyme (t=0) and after 30 min for the NTP synthesis direction (a), c), e), g) and i)) and before the addition of enzyme (t=0) and after 210 min for the polyP synthesis reaction (b), d), f), h) and j) ; mAU, milli-absorption units at 254 nm).

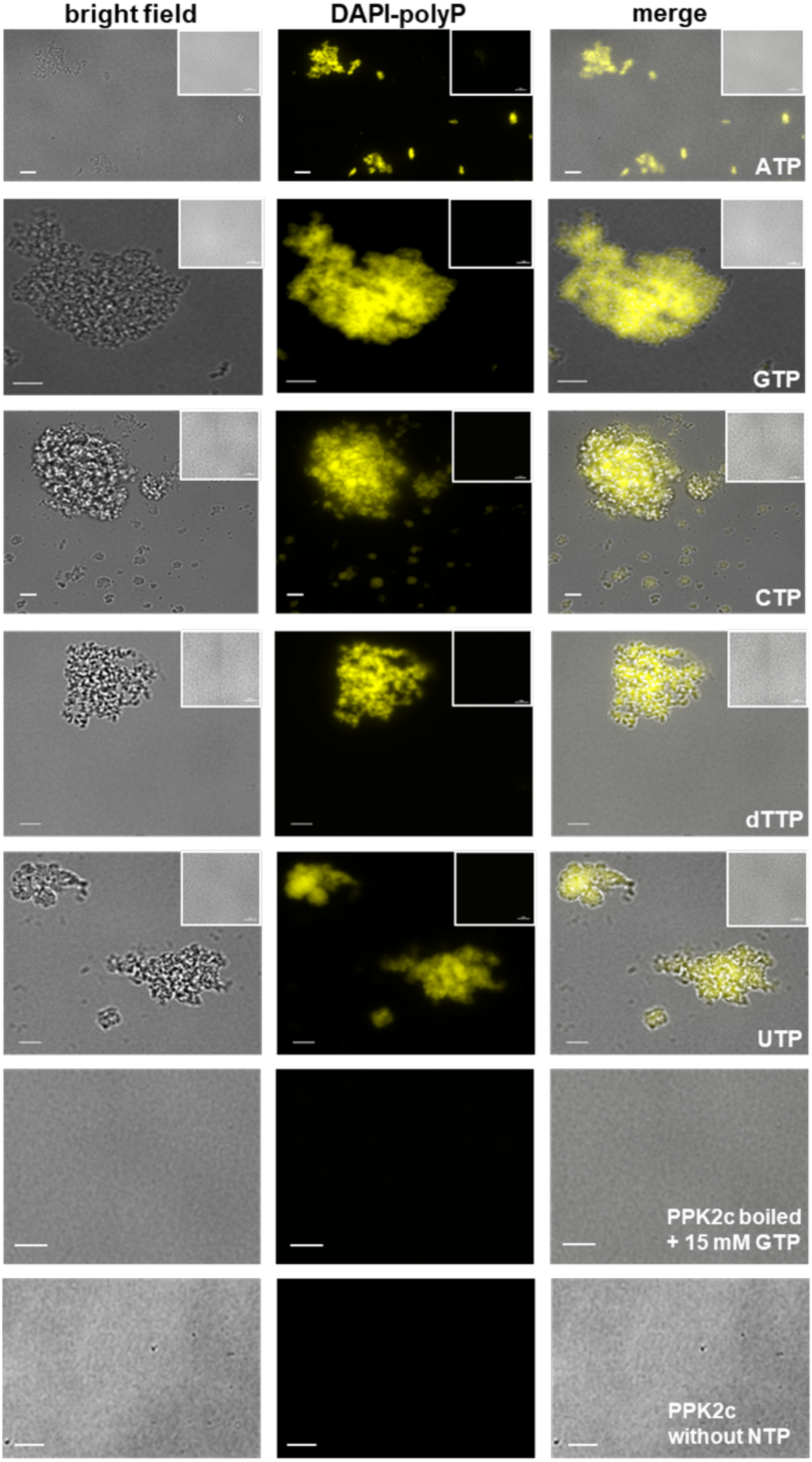

Online Resource 4: *In vitro* formation of polyP granules with purified PPK2c and 15 mM of NTP as indicated. The reaction mixture (10µl) after 30 min incubation at 30°C was stained with DAPI, spotted onto an agarose pad and imaged by fluorescence microscopy. Scale bar 10 µm (or 5 µm in inlays). The inlay figures show the results if PPK2c was omitted.

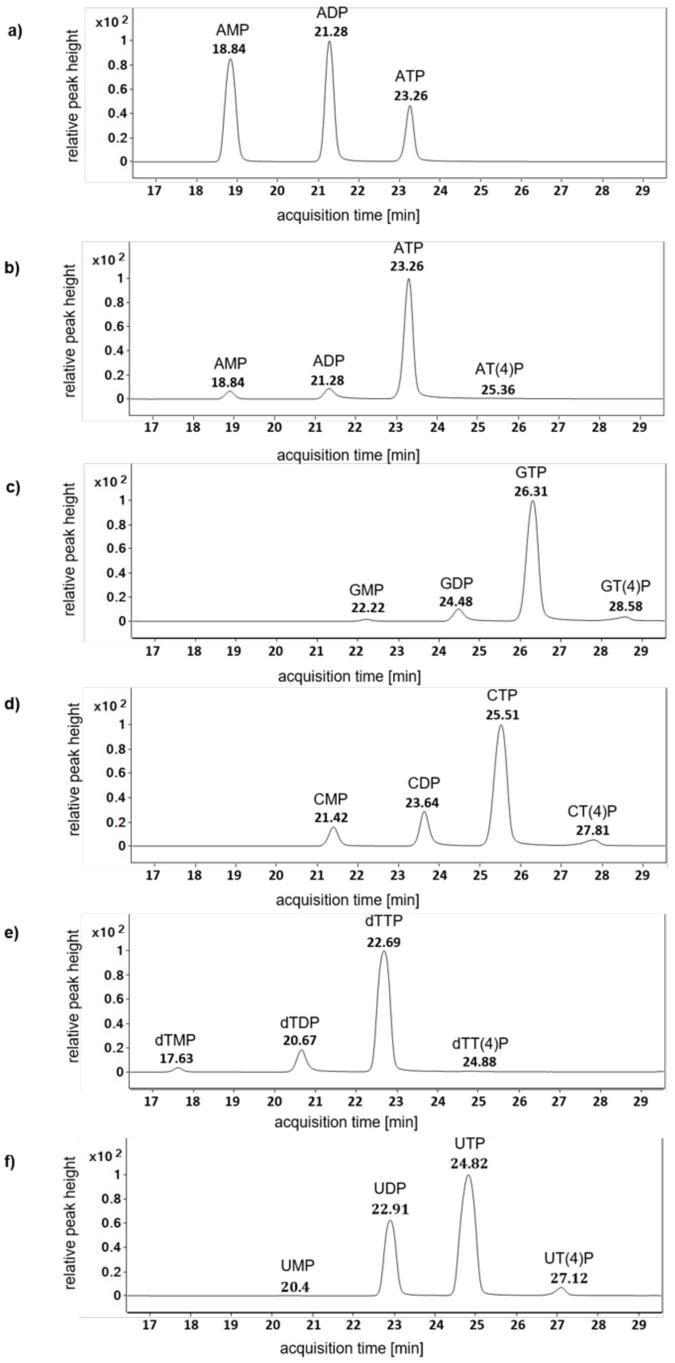

Online Resource 5: HPLC-MS/MS chromatograms of adenosine standard mixtures (a) and PPK2c reaction assays using ADP (b), GDP (c), CDP (d), dTDP (e), and UDP (f) as substrates. Peak heights [%] were normalised to the most abundant peak. Related MRM mass transitions for the targeted detection of corresponding nucleoside phosphates are depicted in **Online Resource 2.**
